# Implementing Single-Pill Combination Therapy for Hypertension: A Scoping Review of Key Health System Requirements in 30 Low- and Middle-Income Countries

**DOI:** 10.5334/gh.1087

**Published:** 2022-01-25

**Authors:** Eleanor Bruyn, Long Nguyen, Aletta E. Schutte, Adrianna Murphy, Pablo Perel, Ruth Webster

**Affiliations:** 1 School of Population Health, University of New South Wales, Sydney, AU; 2 The George Institute for Global Health, University of New South Wales, Sydney, AU; 3 Hypertension in Africa Research Team, MRC Unit for Hypertension and Cardiovascular Disease, North-West University, Potchefstroom, ZA; 4 Centre for Global Chronic Conditions, London School of Hygiene and Tropical Medicine, London, UK; 5 Centre for Health Economics Research and Evaluation, University of Technology, Sydney, AU

**Keywords:** Single-pill combination, antihypertensives, hypertension, low- and middle-income countries, essential medicines list, hypertension treatment guidelines, high blood-pressure

## Abstract

**Objective:**

The World Health Organization (WHO) included single-pill combination (SPC) antihypertensive medications on their 2019 essential medicines list (EML) to encourage uptake and improved hypertension control. We documented key national-level facilitators (SPCs on national EMLs, recommendation for SPCs in national hypertension guidelines and availability of SPCs on the market) supporting uptake of SPCs in the 30 most populous low- and middle-income countries (LMICs).

**Methods:**

A hierarchical information gathering strategy was used including literature and web searches, the use of organisational databases and personal communications with colleagues to obtain information on (1) whether SPC antihypertensives are on national EMLs, (2) whether SPC antihypertensives are recommended in national hypertension guidelines and (3) whether SPCs are available on the market.

**Results:**

Eleven of 30 LMICs had all facilitators in place being Egypt, Kenya, Nigeria, Sudan, China, the Philippines, Thailand, Iran, Argentina, Colombia and Mexico. Twenty-six countries had national hypertension guidelines (or similar) in place with SPCs being recommended in 18 of these. Apart from Afghanistan, SPCs were available on the market in all countries. The facilitator least present was the inclusion of SPC antihypertensives on national EMLs at 12 of 29 (Turkey does not have an EML).

**Conclusion:**

This study demonstrated that many LMICs have made significant progress in their uptake of SPC antihypertensives and several had included SPCs on their EMLs and guidelines prior to their inclusion on the WHO EML. Despite this progress, the uptake of SPC antihypertensives in LMICs could be improved including through their further inclusion on EMLs.

## Introduction

Raised blood pressure is the primary risk factor for cardiovascular disease and mortality worldwide [1,2], responsible for 10.8 million deaths in 2019 [2]. The number of adults with hypertension increased worldwide from 594 million in 1975 to 1.13 billion in 2015 and has transitioned from being a risk factor that predominantly affect high-income countries to one that is now more prevalent and increasing rapidly in low- and middle-income countries (LMICs) with Sub-Saharan Africa, south Asia and central and eastern European regions thought to have the highest prevalence [3,4].

Despite the existence of effective and affordable antihypertensive treatment, hypertension is not always well detected, treated, or controlled in high-income countries but even less so in LMICs [5,6]. In a nationally representative cross-sectional study of 1.1 million adults across 44 LMICs, of those with hypertension (17.5%), only 30% had received treatment and 10.3% had controlled hypertension [5].

Although the majority of patients with hypertension require a combination of 2–3 blood pressure-lowering medications to achieve control [7,8,9], most patients in LMICs still receive monotherapy [10]. The use of single-pill combination (SPC) therapy, also known as fixed-dose combination (single pills that contain a combination of two or more active ingredients) in the management of hypertension is largely accepted as a safe and efficient means of reducing treatment complexity and rapidly improving blood pressure (BP) control [1,7,8,9]. First-line treatment with combination therapy has been associated with a significant (34%) risk reduction of cardiovascular events or all-cause death, when compared to those who received delayed combination treatment initiation due to initial monotherapy treatment [11]. This was primarily due to the more rapid and effective BP control [11]. Combination therapy is also associated with lower healthcare resource use, which is particularly important for LMICs [12].

### Essential Medicines Lists

In 2019, the World Health Organization (WHO) included four SPC antihypertensive medications on its essential medicines list (EML) being lisinopril + amlodipine, lisinopril + hydrochlorothiazide, telmisartan + amlodipine and telmisartan + hydrochlorothiazide [13]. Although a broad range of medications are widely available in most markets for private purchase, EMLs are used by national governments and institutions to determine which medicines warrant being funded, stocked, prescribed and dispensed in public health services, which provide the majority of affordable care [14]. As such, EMLs at the WHO and national levels, can influence the medicines that people have access to and therefore, constitute a significant determinant of health [14].

Access to medicine is a known challenge in all countries, with context specific facilitators and barriers. There is limited data available on whether necessary macro level health system factors are in place in LMICs to support the widespread use of SPC antihypertensives.

We conducted a scoping review of three national-level facilitators likely to affect the implementation of SPC therapy for the treatment of hypertension in the top 30 most populous LMICs. These included:

1. The presence of national EMLs and whether SPC antihypertensives were included;
2. The presence of national hypertension treatment guidelines and whether SPC antihypertensives were recommended; and
3. The availability of both generic and branded SPC antihypertensives on the market in each country.

## Methods

### Research design

This scoping review used a hierarchical information gathering strategy. We considered Bigdeli et al.’s comprehensive framework on access to medicines which details multiple barriers which medications must traverse through the health system before reaching the patient [15], to identify key macro level facilitators including listing of SPCs on national EMLs, recommendations for use of SPCs in national treatment guidelines, and availability of SPCs on the market in each country.

### Country selection and characteristics

We ranked and selected the top 30 LMICs according to their population size based on World Bank data (September 2020) and noted their economic stratum [16,17]. Data on hypertension prevalence for each country was obtained from the WHO Global Health Observatory, using the indicator ‘Raised BP (SBP≥140 and/or DBP ≥90) (age standardized estimate)’ [18]. This data was last updated on 17 November 2017 for estimates as of 2015.

### Search strategy

#### National Essential Medicines Lists

We accessed and reviewed EMLs uploaded to the WHO National Essential Medicines Lists Repository [19]. Due to the age of many documents and the fact that uploading EMLs is a voluntary exercise, we attempted to locate more recent versions through an online search (key words: [country’s name] AND essential medicines list). We also used contacts from Resolve to Save Lives [20], to obtain more up to date EMLs. For countries where we could not find any online evidence of an EML, we contacted colleagues working in those countries for further information.

#### Hypertension treatment guidelines

For national hypertension treatment guidelines we searched several sources including the WHO Essential Medicines and Health Products Information Portal [21], the Hypertension Cardiovascular Outcome Prevention and Evidence in Asia (HOPE Asia) Network [22], and official websites of national hypertension societies. We also used PubMed and Google (key words: [country’s name] AND hypertension treatment guidelines) to confirm and to identify additional guidelines not available elsewhere. The guidelines were reviewed for SPC recommendations if they were in English or able to be translated by commonly available language translation software. For guidelines where translation software was unable to be used, colleagues who were fluent in that language assisted where available.

#### Availability of SPC antihypertensives on the market

We used an online search strategy (key words: [country name] AND ‘online pharmacy’ in either English or translated to local language using translation software) to identify online pharmacy services located in the countries of interest. Where an online pharmacy was found, we searched for common SPC antihypertensives. For countries where online pharmacies were not identified, PubMed and Google searches were used to identify literature that documented the availability of SPC antihypertensives and/or generic versions for sale in each country (key words: [country’s name] and a combination of ‘hypertension’ or ‘antihypertensive’ AND ‘medication’ or ‘medicine’ or ‘drug’ or ‘treatment’ AND/OR ‘generic’). For countries where insufficient information was found online, colleagues working in those countries were contacted.

### Ethics

This study predominantly comprised an online scoping review and document analysis, hence ethics approval was not required.

## Results

The top 30 most populous LMICs spanned five geopolitical regions (***Table 1***), including Africa (11 countries), Asia (10 countries), Europe (2 countries), Latin America (4 countries) and the Middle East (3 countries). Five countries were classified by the World Bank as low-income (LIC), 13 countries were lower-middle-income (LMIC) and 12 countries were upper-middle-income (UMIC) [16]. The total population of these 30 countries was approximately 5.45 billion, or 71% of the global population based on the UN estimate of 7.7 billion in 2019 [17]. Based on age-standardized rates of hypertension prevalence in adults aged 18 and above (***Table 1***), these 30 countries include approximately 1.36 billion people affected by hypertension.

**Table 1:** Status of SPC for the treatment of hypertension across national-level facilitators in the top 30 most populous LMICs.


COUNTRY	POPULATION (WORLD BANK 2019) [17]	SOCIO-ECONOMIC STATUS (WORLD BANK 2020) [16]	PREVALENCE OF HYPERTENSION – AGE STANDARDIZED (WHO 2017) %, [95% CI] [18]	INCLUSION OF SPC ANTIHYPERTENSIVE IN NATIONAL EML (YEAR OF EML PUBLICATION)* IF YES, INCLUDED SPCS ARE LISTED	NATIONAL HYPERTENSION TREATMENT GUIDELINES (YEAR OF PUBLICATION)	INCLUSION OF SPC ANTIHYPERTENSIVES IN NATIONAL TREATMENT GUIDELINES, AND CONTEXT FOR USE	AVAILABILITY ON MARKET OR FOR SALE – INCLUDING DATA SOURCE	AVAILABILITY OF GENERICS – INCLUDING DATA SOURCE

**Africa**								

Algeria	43,053,054	LMIC	25.1% [19.4–31.5]	Yes (2016) Valsartan/HCTZ Losartan/HCTZ Irbesartan/HCTZ Candesartan/HCTZ Quinapril/HCTZ Captopril/HCTZ Enalapril/HCTZ Amiloride/HCTZ Atenolol/Nifedipine Perindopril/Indapamide	Not Available	Not Available	Yes – EML	Yes – online pharmacy [44]

Congo, Democratic Republic	86,790,567	LIC	28.5% [21.2–36.5]	No (2010)	Not Available. Recent literature indicated utilization of WHO/ISH (2003)[29]	Not Available	Yes – online pharmacy [45]	Yes – online pharmacy [45]

Egypt, Arab Republic	100,388,073	LMIC	25% [19.8–30.6]	Yes (2012–2013) Lisinopril/HCTZ	Yes (2014)[46]	Yes. Combination therapy (SPC as an option) if monotherapy fails in low-immediate risk groups. Combination therapy as initial treatments in high and very high risk groups.	Yes – EML	Yes – online pharmacy [47]

Ethiopia	112,078,730	LIC	30.3% [23.1–38]	No (2015) [48] No (EPSA Pharmaceutical list 2020)	Yes (2016) – as part of guidelines for multiple conditions [49]	Yes. Combination therapy if monotherapy fails. Combination therapy (DHCCB + ACEI) as initial treatments if BP>160/100. SPC not explicitly mentioned.	Yes – national formulary 2007 [50] + literature [51]	Unsure

Kenya	52,573,973	LMIC	26.7% [20.2–34]	Yes (2019) [52] Amlodipine/HCTZ Telmisartan/HCTZ Losartan/HCTZ Lisinopril/HCTZ Telmisartan/Amlodipine	Yes (2018) – as part of guidelines for CVD management, adapted from ESH/ESC 2013 [53]	Yes. Combination therapy (SPC as an option) as second line if monotherapy fails in level 1 hypertension. As first-line treatment for level 2 hypertension and above [53]	Yes – EML + national guidelines + online pharmacy [54]	Yes – online pharmacy [54]

Morocco	36,471,769	LMIC	26.1% [20–32.9]	No (2017)	Not Available	Not Available	Yes – literature [55]	Unsure – although literature indicated significant increase in generic usage in general [56]

Nigeria	200,963,599	LMIC	23.9% [18.7–29.5]	Yes (2016) Reserpine/dihydroergocristine/clopamide	Yes (2005) as per literature [34,35]	Yes – to improve adherence but unclear position in guideline	Yes – EML + national guidelines + online pharmacy [57] + literature [35]	Yes – online pharmacy [57]

South Africa	58,558,270	UMIC	26.9% [21.7–32.7]	No (2018)	Yes (2014) [58]	Yes – Recommend starting with combination treatment if BP ≥ 160/100 and can be considered for all others as well. SPCs recommended due to improved adherence and BP control.	Yes – EML + national guidelines + literature [59]	Yes – literature [59]

Sudan	42,813,238	LIC	30.2% [23–37.8]	Yes (2014) [60] for 2014–2016 period Amlodipine/ Valsartan Candesartan/HCTZ	Yes (2014) – as part of national guidelines for multiple conditions [61].	Unsure – Combination therapy, but not SPC specifically, recommended as second line treatment.	Yes – EML	Yes – professional contact

Tanzania	58,005,463	LMIC	27.3% [21.4–33.8]	No (2017)[62]	Yes (2017) – incorporate into national EML along with guidelines for multiple conditions [62].	Unsure – Combination therapy, but not SPC specifically, recommended as second line treatment [62].	Yes – literature [63]	Yes – professional contact

Uganda	44,269,594	LIC	27.3% [21–34.2]	No (2016)	Yes (2016) – as part of national guidelines for multiple conditions [64].	Unsure – Combination recommended but not SPC specifically.	Yes – online pharmacy [65]	Yes – online pharmacy [65]

**Asia**								

Afghanistan	38,041,754	LIC	30.6% [23.6–38.3]	No (2014)	Yes (2013) – as part of national guidelines for multiple conditions [66].	Unsure – Combination recommended but not SPC specifically. Not recommended for first-line treatment [67].	Unsure	Unsure

Bangladesh	163,046,161	LMIC	24.7% [19.1–30.6]	No (2018)^&^	Yes (2013) [68]	Yes. Stage 1 hypertension: combination therapy is recommended if monotherapy fails. SPC recommended to improve compliance Stage 2 hypertension: combination therapy as standard initial treatment.	Yes – national guidelines + online pharmacy [69]	Yes – online pharmacy [69]

China	1,397,715,000	UMIC	19.2% [14.9–24]	Yes (2019)^&^ Amlodipine/Benazepril Benazepril/HCTZ Lisinopril/HCTZ Olmesartan/HCTZ Irbesartan/HCTZ Losartan/HCTZ Telmisartan/HCTZ Valsartan/HCTZ Valsartan/Amlodipine Olmesartan/Amlodipine Telmisartan/Amlodipine Perindopril/Indapamide Perindopril/Amlodipine	Yes (2018)[70]	Yes. Combination therapy (including SPC) recommended for high risk groups with BP ≥ 160/100 mmHg and 20/10 mmHg higher than the target BP or those where monotherapy is inadequate. Low dose SPC can be initiated in those with BP ≥ 140/90 mmHg.	Yes – EML + national guidelines	Yes – professional contact + online news article [71]

India	1,366,417,754	LMIC	25.8% [21.3–30.7]	No (2015)	Yes (2016)[72]	Yes – combination therapy recommended for Grade 3 hypertension, and for Grade 1 and 2 uncontrolled on monotherapy. SPCs recommended once patient is stabilised.	Yes – national guidelines + literature [73]	Yes – online pharmacy [74]

Indonesia	270,625,568	UMIC	23.8% [18.5–29.5]	No (2017)^&^	Yes (2019)[75]	Yes^&^ – combination therapy recommended to be used for initiation of therapy in most patients with use of SPC where available.	Yes – national guidelines + literature [76]	Yes – online pharmacy [77]

Myanmar	54,045,420	LMIC	24.6% [18.5–31.1]	No (2016)	Not available [30]	Not available	Yes – online pharmacy [78]	Yes – online pharmacy [78]

Pakistan	216,565,318	LMIC	30.5% [24.4–37.4]	No (2018)	Yes (2018)[79]	Yes – recommended to use SPC as much as possible and as early as possible.	Yes – EML+ national guidelines + online pharmacy [80]	Yes – online pharmacy [80]

Philippines	108,116,615	LMIC	22.6% [17.4–28.1]	Yes (2017) Enalapril/HCTZ Irbesartan/HCTZ Losartan/HCTZ Telmisartan/HCTZ Valsartan/HCTZ	Yes (2019) as per literature [33]	Yes – SPCs increasingly used since 2013, however monotherapy still the predominant treatment modality.	Yes – EML+ literature [33]	Yes – online pharmacy [81]

Thailand	69,625,582	UMIC	22.3% [16.9–28.3]	Yes (2020) Amiloride/HCTZ	Yes (2019) [82]	Yes – SPC recommended for most. Monotherapy is recommended for weak elderly patients with relatively low initial BP of 140–149/90–99 mmHg and for low-risk patients [82].	Yes – EML + national guidelines + literature [83]	Yes – professional contact

Vietnam	96,462,106	LMIC	23.4% [18–29.4]	No (2017)	Yes (2018)[84]	Yes – SPCs recommended as standard initial treatment.	Yes – national guideline + online pharmacy [85]	Yes – online pharmacy [85]

**Europe**								

Ukraine	44,385,155	LMIC	27.1% [20.7–34.2]	No (2017)^&^ [86]	Yes (2012) mentioned in literature [32]	Unsure (Couldn’t find or access the actual guidelines).	Yes – literature [87]	Yes – literature [88]

Russia	144,373,535	UMIC	27.2% [21.2–33.6]	No (2014)^&^	Yes (2019) [89]	Yes – Combination therapy (SPC to improve adherence) is initial therapy in most patients. Low dose combination preferred over maximum dose monotherapy. (*translated by professional contact).	Yes – national guidelines + literature [90]	Yes – literature [91]

**Latin America**								

Argentina	44,938,712	UMIC	22.6% [17–28.9]	Yes (2010)^&^ Amiloride/HCTZ	Yes (2018)^&^ [92]	Yes – SPC recommended for most – monotherapy as first-line treatment is only recommended for with low CVD risk and level 1 hypertension.	Yes – EML + national guidelines + literature [93]	Yes – online pharmacy^&^ [94]

Brazil	211,049,527	UMIC	23.3% [18.1–28.8]	No (2017)^&^	Yes (2016)[95]	Yes – Stage 1 + low and intermediate CVD risk: combination therapy if monotherapy fails. Stage 1 + high CVD risk, Stages 2 and 3: dual combination therapy as standard initial treatment. SPC as an option to improve adherence.	Yes – national guidelines + literature [96]	Yes – online pharmacy^&^ [97]

Colombia	50,339,443	UMIC	19.2% [14.2–24.7]	Yes (2011)^&^ Losartan/HCTZ	Yes (2017)^&^ [98]	Yes – SPC recommended for those with BP greater than 160/100 mmHg and with risk characteristics [98]	Yes – EML + national guidelines	Yes – online pharmacy^&^ [99]

Mexico	127,575,529	UMIC	19.7% [14.8–25.1]	Yes (2011)^&^ Candesartan/HCTZ Losartan/HCTZ	Yes (2014) [100]	Unclear^&^ – combination therapy recommended when uncontrolled on monotherapy, or for first line treatment is BP >20/10 mmHg above target. However, SPCs not specifically mentioned.	Yes – EML	Yes – online pharmacy^&^ [101]

**The Middle East**								

Iran, Islamic republic	82,913,906	UMIC	19.7% [15.2–24.6]	Yes (2014) Amiloride/HCTZ Valsartan/Amlodipine Valsartan/Amlodipine/HCTZ Lisinopril/HCTZ Losartan/HCTZ Triamterene/HCTZ Valsartan/HCTZ	Yes (2015) [102]	Unclear – Second line where monotherapy is inadequate OR as initial treatment where BP is >= 20mmHg systolic or >= 10mmHg diastolic above target. SPCs not specifically mentioned.	Yes – EML	Yes – professional contact

Iraq	39,309,783	UMIC	25.2% [19.1–31.6]	No (2010)	Yes (2012)[103]	Unsure – Combination recommended but not SPC specifically.	Yes – literature [104]	Yes – professional contact

Turkey	83,429,615	UMIC	20.3% [15.9–24.9]	No EML on portal OR in 2014 review [23]	Yes (2019)[105]	Yes^&^ – either monotherapy or combination therapy is recommended for treatment initiation. SPCs are recommended for improving patient adherence.	Yes – national guidelines + literature [106]	Unsure


Note: If SPCs were listed on the national EML, or specially recommended in guidelines, it was assumed that they were available on the market in that country. ESH/ESC = European Society of Hypertension/European Society of Cardiology WHO/ISH = World Health Organization/International Society of Hypertension EML = Essential Medicines List SPC = Single-pill combination HCTZ = Hydrochlorothiazide LIC = Low-Income Country LMIC = Lower-Middle-Income Country UMIC = Upper-Middle-Income Country CVD = Cardiovascular Disease BP = Blood Pressure DHCCB = Dihydropyridine Calcium Channel Blocker ACEI = Angiotensin Converting Enzyme Inhibitor EPSA = Ethiopian Pharmaceuticals Supply Agency *EML is available from the WHO National Essential Medicines List Repository [19], unless referenced otherwise. &Document not in a language where the investigators had access to a native speaker and has been interpreted using translation software.

### National EMLs and inclusion of SPCs

We were able to locate national EMLs for 28/30 countries (Turkey and Tanzania being the exceptions) from the WHO EML repository [19]. Through an online search, we were able to locate a national EML for Tanzania and more up to date EMLs for Kenya, Sudan, Ukraine and Ethiopia. We were able to access more recently updated national EMLs for China, the Philippines, Thailand and Vietnam through Resolve to Save Lives. There was no national EML that could be located for Turkey and research indicated its non-existence as of 2014 [23]. See Supplemental Digital Content 1 (SDC 1), which illustrates the data source for the most up to date national EMLs, national hypertension guidelines and availability of single-pill combination antihypertensives.

**Figure 1:**
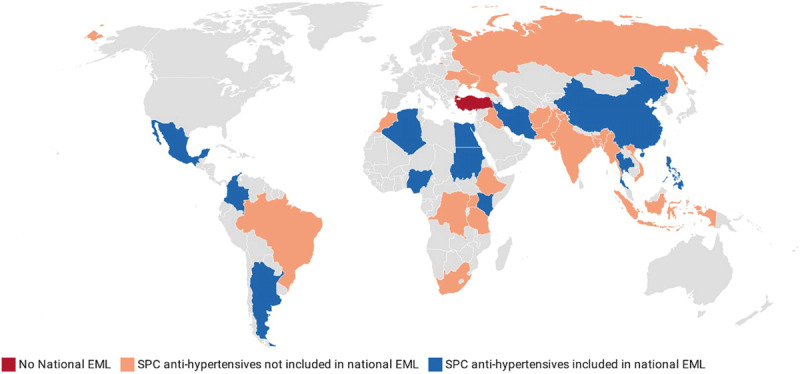
Availability of national essential medicines lists (EML) and inclusion of SPCs for the treatment of hypertension among the top 30 most populous low- and middle-income countries.

Twelve countries (40%) included some form of SPC antihypertensives on their national EMLs, with these countries covering several geopolitical regions and a broad range of economic strata (***Table 1, Figure 1***). Identified national EMLs were dated between 2010 to 2020 with a median publication year of 2016. There was no clear relationship between the year of the EML being updated and whether SPC antihypertensives were included on that EML. The most common forms of SPCs listed included Angiotensin Converting Enzyme Inhibitors (ACEI) + Hydrochlorothiazide (HCTZ), Angiotensin Receptor Blocker (ARB) + HCTZ, calcium channel blocker (CCB) + ARB and Amiloride + HCTZ combinations.

### Hypertension treatment guidelines

#### Inclusion of SPCs in international guidelines

Many LMICs use international hypertension treatment guidelines or base their national hypertension guidelines on these [24]. The WHO ‘Guideline for the pharmacological treatment of hypertension in adults’ (published 24 August 2021) recommends combination therapy, preferably in the form of SPCs for initial treatment in adults [25].

The International Society of Hypertension 2020 guidelines recommend dual low dose SPCs as the optimal initial treatment for hypertension with the exception of ‘low risk grade 1 hypertension or in very old (≥ 80 years) or frailer patients’ who should be considered for monotherapy [26]. Similarly, the European Society of Cardiology 2018 guidelines recommend initial therapy with dual combination of ACEI or ARB with CCB or diuretic, preferably as SPC, with the same aforementioned recommendations of monotherapy in certain groups [27]. The 2017 American College of Cardiology/American Heart Association guidelines support initiation of dual combination therapy as either separate agents or SPCs in adults with grade 2 hypertension (defined as SBP ≥ 140mmHg and/or DBP ≥ 90mmHg) and an average BP > 20/10mmHg above their BP target [28].

#### Inclusion of SPCs in national guidelines

National hypertension guidelines were identified for most countries (26/30) (***Figure 1***, SDC 1). We were unable to confirm the existence of guidelines for either Algeria or Morocco; the Democratic Republic of Congo and Myanmar do not currently have official national hypertension treatment guidelines [29,30]. For Nigeria, Ukraine, and the Philippines, national guidelines exist, however, we were unable to obtain the official documents for review [31,32,33].

Of the 23 countries with national hypertension treatment guidelines available for review, 18 countries specifically recommended SPC antihypertensives, with the majority recommending them for second-line treatment if monotherapy is inadequate in lower risk patients to improve adherence or as an initial option in those with higher risk/elevated BP (above 160/100mmHg, or systolic pressure ≥ 20mmHg/diastolic pressure ≥ 10mmHg above target BP) (***Table 1***). Notably, five countries (Indonesia, Pakistan, Russian Federation, Turkey and Vietnam), recommend SPCs as initial treatment regardless of BP level. Eight national guidelines did not explicitly recommend SPCs but did recommend combination therapy. In Nigeria and the Philippines, SPCs were recommended but we were unable to confirm in what context due to being unable to source the actual guidelines [33,34,35]. The guidelines were dated from 2012 to 2019 with a median publication year of 2016.

### Availability of SPC antihypertensives on the market

Data on the presence of SPCs on the market, including generic options, was obtained primarily through online and literature searches (SDC 1). For all countries, except for Afghanistan, we were able to document availability with twenty countries having evidence of both branded and generic SPC antihypertensives (***Table 1***). Professional contacts also confirmed availability of generic SPC antihypertensives in an additional six countries. For four countries (Ethiopia, Morocco, Afghanistan and Turkey), we were unable to identify whether generic brands of SPC antihypertensives were available.

### Overall availability of national-level facilitators

**Figure 2:**
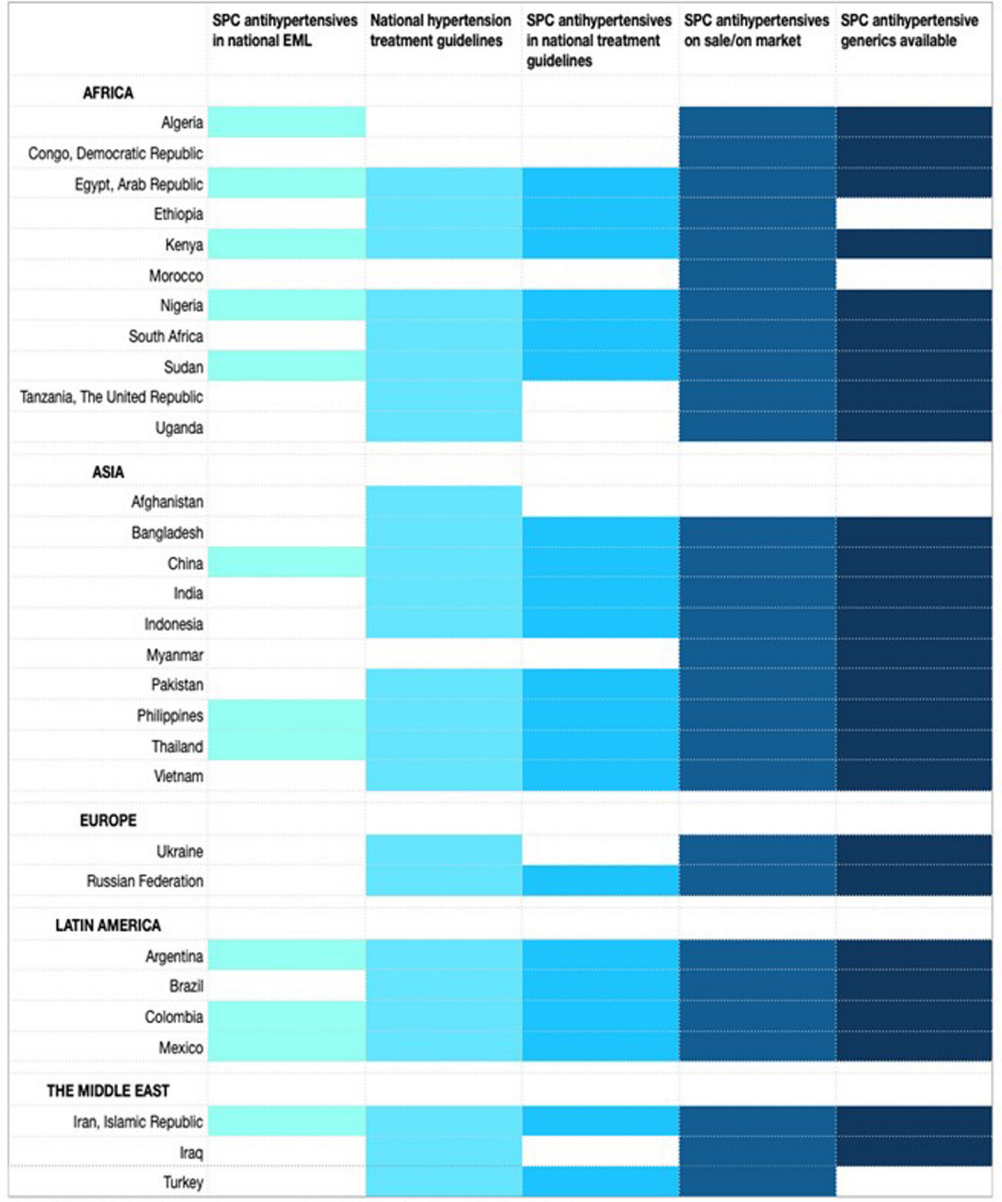
Prescence of national-level facilitators for the uptake of SPC antihypertensives in the 30 most populous LMICs. *Notes*: Coloured block indicates the presence of the particular national level facilitator in that country. SPC = Single Pill Combination. EML = Essential Medicines List.

Overall, 11 countries (37% – Egypt, Kenya, Nigeria, Sudan, China, the Philippines, Thailand, Argentina, Colombia, Mexico and Iran) had all national-level facilitators in place including (1) presence of a national EML with SPC antihypertensives included, (2) the presence of national hypertension treatment guidelines with recommendations for use of SPC antihypertensives, and (3) availability of SPC antihypertensives on the market including generics (***Figure 2***). Most of the remaining countries had at least one facilitator in place but 16/28 countries (57%) were primarily missing the inclusion of SPCs on national EMLs.

## Discussion

Despite the advantages of SPC antihypertensive medications in improving BP control, only 11 of the 30 countries (37%) under review had all national-level facilitators in place to ensure uptake (***Figure 2***). These countries are geographically diverse with a combined population of approximately 2.3 billion people and represent a range of income levels: one LIC, four LMICs and six UMICs.

### International policy context

International policies, especially those developed by the WHO, influence how LMICs develop their own [15,26]. WHO endorsement of four combinations of SPC antihypertensive medication is expected to provide confidence to LMICs to include such listings on their own national EMLs [36]. International treatment guidelines, including the WHO’s recently published (24 August 2021) ‘Guideline for the pharmacological treatments of hypertension in adults’ also have the capacity to influence local treatment guidelines and/or impact prescriber practice [25]. International policy, incorporating international treatment guidelines as well as market availability and inclusion on the WHO EML, is thus now well aligned to support the use of SPC antihypertensives in the management of hypertension. The WHO and international organisations including the American, European and International Societies of Hypertension have taken significant steps in recommending the use of SPC antihypertensives and now it is up to national governments to translate this into local implementation.

While the WHO has taken important steps to recommend the use of SPC antihypertensives through their EML and most recently, through their guidelines, the absence of such resources and formal endorsement in the past may have contributed to delays in SPC uptake. This is particularly true for LMICs, who may rely on WHO guidance when implementing policy changes.

### National EMLs and inclusion of SPCs

We were able to access a national EML for 29/30 of the most populous LMICs, with the exception being Turkey. Of the national EMLs that we could access, 12 included SPC antihypertensives. SPC antihypertensives were included in the WHO EML in 2019, but many of the national EMLs we accessed were published prior to 2019, indicating several countries had included SPCs even before WHO listed them, but also indicating that country EMLs may not have had the opportunity to incorporate SPCs following listing by the WHO. If SPC antihypertensives are not included on a national EML, it is unlikely that they will be widely available in the public health system. However, inclusion of a medication on the WHO EML alone is not sufficient to affect uptake in LMIC settings [37], as implementation must be part of a larger strategy endorsed by the Ministry of Health with the aim of improving availability, affordability, accessibility, and medicine adherence, with the design of suitable health system delivery models [37].

### Hypertension treatment guidelines

Of the 30 countries included in this study, only 26 countries had national hypertension treatment guidelines or primary care guidelines with hypertension included. For certain presentations of hypertension, SPC antihypertensives were specifically recommended in 18 of the guidelines. A further eight guidelines recommended combination therapy for the treatment of hypertension but did not mention SPCs specifically.

Most patients with hypertension in LMICs receive monotherapy with relatively few on combination therapy, and even fewer on SPCs [10]. In a cross-sectional study on hypertension in HICs to LICs with more than 140,000 participants, the use of two or more medications was 18.1% in HICs (95% CI, 17.2%-19.1%), but 14.1% in LMICs (95% CI, 13.7%-14.6%) and only 1.6% in LICs (95% CI, 1.4%-1.8%) [10]. This aligns with many of the national hypertension treatment guidelines in LMICs, which tend to recommend monotherapy as first-line treatment (see ***Table 1***). Where SPC antihypertensives are recommended as first-line treatment, it is predominantly for those with grade 2 hypertension, those with comorbidities and those with cardiovascular risk factors.

National EMLs and hypertension treatment guidelines provide guidance to the health sector to ensure national consistency in access to medicines and to improve hypertension control.

The inclusion of SPC antihypertensives in these guidelines could facilitate improvement in access through influencing prescribing practices [38]. However, whilst an important next step, guidelines are but one factor and are unable to change uptake and availability independently.

### Availability of SPC antihypertensives on the market

SPC antihypertensives are available in all countries under review (except Afghanistan), indicating good market access. Market availability is a significant initial step in ensuring accessibility to consumers as market forces are a key determinant of access to medicines [10]. It indicates that a regulatory pathway exists for approval of SPC antihypertensives as well as market interest from pharmaceutical companies in providing such products but does not equate to equitable access and availability.

### Availability of generic options

Generic options of SPC antihypertensives were available in 26/30 countries. This is significant as medicine affordability is a key determinant of access to medicines, and relevant to LMICs where health budgets are limited. This is despite there being only 12 of the 30 countries with SPC antihypertensives included on their national EML and demonstrates active market forces despite a lack of policy support. The availability of generic options for SPC antihypertensives, combined with associated financing and subsidisation schemes would improve affordability.

### Strengths and Limitations

This study is the first scoping review we are aware of which documents macro level health system factors that may facilitate the uptake of SPC antihypertensives in LMICs. Strengths of this study include coverage of the top 30 most populous LMICs, covering 71% of the global population and over a billion patients with hypertension. Whilst the decision to include the 30 most populous LMICs covers much of the world’s population, they only represent 22% of the total number of LMICs (135 as of 2020) [39]. As a result, our findings may not be generalizable to other LMICs.

Identifying and sourcing relevant documents was challenging and time consuming and it has been difficult to ascertain the most recent EMLs and hypertension treatment guidelines which has potential implications for the currency of our data and consequentially, our interpretation.

Whilst this study includes several facilitators, they are limited to national policy contexts and the supply chain so do not encompass the complexity of health system factors affecting access to SPC antihypertensives including at the community, household and individual levels [10]. For example, physician awareness and adherence to hypertension treatment guidelines has been consistently documented to be highly variable [10,40,41], and so are patient adherence to hypertension treatments in general [42,43]. Multiple other more complex and variable factors may include socio-political context, health system development and maturity, quality of health services and medication, pharmaceutical context and prescriber education and behaviour etc. We acknowledge that the factors we studied form a component of complex health systems impacting the use of SPC antihypertensives and do not indicate widespread accessibility or affordability. As previously noted however, these policy contexts can facilitate the availability and use of SPC antihypertensives.

### Complexity of implementation challenges

While distinct, each aspect of the health system needed to successfully improve patient access to antihypertensive SPCs does not exist in isolation. They are mutually dependent and are influenced by the broader context of each country including culture, history, economic development and health system advancement, etc. Many countries in this study are adversely affected by ongoing wars or civil unrest, corruption, poverty, under-developed economies, and other competing public health challenges. Due to such heterogeneity, each LMIC requires a unique multifactorial approach that not only advances their hypertension management strategy but also other influential factors within the health system and evidently the holistic development of the country.

## Conclusion

Market access and international policy support for the use of SPC antihypertensives is strong. There is evidence of widespread market availability of SPCs (including generics) in LMICs, but availability alone is not sufficient as supportive national policy is key. National hypertension treatment guidelines do not always align with international policy as monotherapy appears to be the dominant treatment strategy, and national EMLs do not include SPC antihypertensives in the majority of LMICs we reviewed. Opportunities for demonstrating the value of SPCs in hypertension management may include case studies of those countries with established national-level facilitators. Further research into the meso and micro level factors influencing the uptake of SPC antihypertensives may produce further learnings to support national governments, the health sector and industry (among others) in improving access to SPC antihypertensives.

## Additional File

The additional file for this article can be found as follows:

10.5334/gh.1087.s1 Supplemental Digital Content 1 (SDC 1). Table which illustrates the data source for national essential medicine lists, national hypertension treatment guidelines and availability of single-pill combination antihypertensives.
