# Supplementary material for: Implementing Single-Pill Combination Therapy for Hypertension: A Scoping Review of Key Health System Requirements in 30 Low- and Middle-Income Countries
Source: Glob Heart. 2022 Jan 25;17(1):6. doi: 10.5334/gh.1087 (PMC8796691; doi:10.5334/gh.1087)
Supplement: Supplemental Digital Content 1 (SDC 1). — Table which illustrates the data source for national essential medicine lists, national hypertension treatment guidelines and availability of single-pill combination antihypertensives. [file gh-17-1-1087-s1.pdf]

Implementing single-pill combination therapy for hypertension: a scoping review of key health system requirements in 30 low- and middle-income countries

SUPPLEMENTAL DIGITAL CONTENT

Supplementary Table 1. Source of data

Data Source	Most updated and accessible national EML	National hypertension guidelines (or general guidelines incorporating hypertension management)	Availability of SPC antihypertensives	Availability of SPC antihypertensive generics
World Health Organisation repository	20 countries	2 countries	12 countries - implicated by inclusion in EML	-
Resolve to Save Lives	4 countries	-	-	-
Online search (including online pharmacy)	5 countries	21 countries + Myanmar (no national guidelines)	8 countries	18 countries
PubMed	-	3 countries + Democratic Republic of Congo (no national guidelines)	15 countries	3 countries
Other professional contacts	-	-	-	6 countries

EML= Essential medicines list
SPC = Single-pill combination
